# Examining Crisis Communication Using Semantic Network and Sentiment Analysis: A Case Study on NetEase Games

**DOI:** 10.3389/fpsyg.2022.823415

**Published:** 2022-02-04

**Authors:** ShaoPeng Che, Dongyan Nan, Pim Kamphuis, Shunan Zhang, Jang Hyun Kim

**Affiliations:** ^1^Department of Human-Artificial Intelligence Interaction, Sungkyunkwan University, Seoul, South Korea; ^2^Department of Interaction Science, Sungkyunkwan University, Seoul, South Korea

**Keywords:** mobile game, crisis communication, semantic network analysis, sentiment analysis, Sina Weibo

## Abstract

The mobile game “Immortal Conquest,” created by NetEase Games, caused a dramatic user dissatisfaction event after an introduction of a sudden and uninvited “pay-to-win” update. As a result, many players filed grievances against NetEase in a court. The official game website issued three apologies, with mix results, to mitigate the crisis. The goal of the present study is to understand user feedback content from the perspective of Situational Crisis Communication Theory through semantic network analysis and sentiment analysis to explore how an enterprise’s crisis communication strategy affects users’ attitudes. First, our results demonstrate that the diminishing crisis communication strategies (excuse and justification) do not change players’ negative attitudes. It was not a failure because it successfully alleviated the players’ legal complaints and refocused their attention on the game itself. Second, the rebuild (apology & compensation) strategy was effective because it significantly increased the percentage of positive emotions and regenerated expectations for the game. The litigation crisis was identified within gamer communications with respect to Chinese gaming companies for the first time. Nevertheless, this does not indicate an increase in overall legal awareness among the larger Chinese population. It may only reflect greater legal awareness among Chinese online gamers. Fourth, gamers emphasized that they and enterprises should be equally involved when communicating with each other. Finally, in-game paid items should be reasonably priced, otherwise, they will drive users to competitors.

## Introduction

With the outbreak of COVID-19, China has paid unprecedented attention to crisis communication *via* social media (Chen et al., 2020), prioritizing it as a tool for crisis communication in the face of major public health emergencies (Su et al., 2021). Moreover, the epidemic has prompted people to switch from offline communication to online or remote communication when dealing with disputes related to products or enterprises (Paska, 2021). Therefore, enterprises have to do so (Obrenovic et al., 2020; Flemming et al., 2021).

Enterprises use crisis communication on social media to maintain their reputation and market position (Utz et al., 2013; Bratu, 2019). Therefore, enterprises must evaluate whether crisis communication strategies have the expected impact on users. In 2021, academic studies on social media crisis communication mainly focused on communication strategies (Triantafillidou and Yannas, 2020; Wang et al., 2021) and information transmission modes (Mirbabaie et al., 2021; Puyod and Charoensukmongkol, 2021). However, there were few studies on whether crisis communication strategies of enterprises can affect users’ attitudes (Stieglitz et al., 2015; Mirbabaie and Zapatka, 2017).

User’s attitude refers to the individual’s proclivity to appraise the goal. For example, the studies of users’ attitudes toward vaccines (Gao et al., 2021), bitcoin (Mnif et al., 2021), social apps (Kocakoç and Özkan, 2022), the liberating movement of COVID-19 (Haupt et al., 2021), and other fields have proved the effectiveness of using social media content to explore user attitudes. Therefore, based on social media content, this study attempts to evaluate the influence of crisis communication tactics on user perceptions.

In the study of the attitude of users in crisis communication, Cho et al. (2021) used SNA to examine how racial concerns were portrayed in Twitter posts posted in English and Chinese, particularly how social media users blamed those responsible for the current crisis. Based on SCCT, Park and Park (2020) examined the public’s opinion on Samsung’s crisis communication strategy through SNA. These studies suggest that SNA can identify users’ attitudes or perceptions on social media. Therefore, this study first uses SNA to explore how an enterprise’s crisis communication strategy affects users’ attitudes.

RQ1. What were the reactions of users after NetEase issued three statements?

Although SNA plays an essential role in inferring the word clusters or framework, it is not easy to measure public emotions quantitatively. Therefore, to address the limitations of SNA, Stieglitz and Krüger (2011) analyzed tweets related to the 2010 Toyota crisis using Linguistic Inquiry and Word Count to categorize sentiment, allowing them to observe the changing trend of positive and negative public sentiments with the release of official declarations. Furthermore, Stieglitz et al. (2018) examined Volkswagen’s exhaust emission scandal and analyzed the mood and content of each period of that scandal. The study reported that Volkswagen’s tweets failed to soothe the negative emotions of consumers. These studies show that sentiment analysis has a noticeable effect on sentiment judgment. Therefore, sentiment analysis is used in the second step of this study to measure the impact of enterprises’ crisis communication strategies on users.

RQ2. How did users’ feelings change after NetEase released each statement?

To sum up, this study is based on the Situational Crisis Communication Theory (SCCT) and the crisis event that “Immortal Conquest,” a game subordinated to NetEase Games, encountered in the Chinese market in 2020. Furthermore, this paper uses Semantic Network Analysis (SNA) and sentiment analysis to explore how enterprises’ social media crisis communication strategies affect users’ attitudes.

We first used the SCCT model to classify the crisis communication strategies adopted by NetEase and then used SNA to analyze user comment texts after the release of official statements to understand the connection between critical issues in the crisis. Finally, sentiment analysis was used to judge the positivity and negativity of these texts. Since NetEase officially issued three statements, the changes of text content and emotional polarity in the comments are the keys to evaluating whether crisis communication strategies impact users’ attitudes.

## Literature Review

### Case Background

This paper analyzes the impact of enterprises’ crisis communication strategies on users’ attitudes in the aftermath of user complaints regarding the game Immortal Conquest, a free-to-play strategy war game developed and operated by the Chinese company NetEase. On May 27th, 2020, the company pushed a controversial update to the game, which implemented deeper integration of a Treasure system that allowed players to enhance their game experience using microtransactions. Many players voiced their concerns before the update, citing the likelihood of a vast “pay-to-win” trend that would not be limited to cosmetic upgrades. Indeed, the update put corporate income over the users’ experience, making it nearly impossible to compete in a fair setting. After the update, players amplified their voices of dissatisfaction on social media. Gamers arranged boycotts in several forms, and some users sued the company in the Court of Hangzhou. NetEase released three apology statements (see Table 1) on the official Immortal Conquest page on Sina Weibo, a Chinese microblogging site, attempting to control the damage.

**Table 1:** Contents and classification of NetEase’s three statements.

	First statement	Second statement	Third statement
Contents	Please give us more time	NetEase provided a deeper explanation of the “Treasure System”	NetEase offered suggestions and solutions to user problems
Strategies	*Diminishing*	*Diminishing*	*Rebuild*
Explanations	(d) *Excuse* (NetEase minimize organizational responsibility by denying the intent to cause harm)	(e) *Justification* (NetEase minimize perceived damage caused by crises)	(f) *Compensation*, (g) *Apology* (NetEase offered gifts to users and said that the organization took full responsibility for the crisis and asked for forgiveness from users)

### Crisis Communication

Crisis communication refers to the continuous behavior and process of crisis resolution and crisis avoidance through communication (Seeger, 2006). It is regarded as an effective way to reduce the impact of a crisis on companies and to enhance user welfare. If a company handles it correctly, it can turn a crisis into an opportunity that the company can take advantage of (Ma et al., 2020). On the contrary, a lack of crisis communication can amplify the crisis causing severe damage or even a collapse of the organization.

Crisis communication is critical in crisis management. According to academic study, crisis communication theories primarily focus on the development of solutions, which aim to reduce the responsibility of organizations or individuals and help them tide over difficulties. For example, according to the image repair theory (Benoit, 2015), after the crisis, the main communication strategies of organizations include *denial strategy*, *evading responsibility*, *reducing offensiveness*, *corrective action,* and *mortification*. SCCT, which is based on attribution theory in social psychology and places defense tactics in stakeholder attribution, is another important theory in this subject (Coombs, 2007a). First, based on the public’s perception of crisis attribution responsibility, SCCT divides crisis into *victim cluster*, *accidental cluster*, and *intentional cluster*. Furthermore, the crisis response strategy is divided into *deny strategy*, *diminishing strategy*, and *rebuild strategy;* Finally, the crisis situation is matched with the coping strategy. According to SCCT, the most effective crisis communication strategy matches an organization’s rhetorical skills to the reputational threat (Coombs and Holladay, 2002).

In a word, crisis communication is studied to help enterprises maximize the probability of reaching a turning point in their crises and minimize the negative impact on consumers.

### Semantic Network Analysis

Semantic network analysis is a method for representing knowledge in graphs in an organized manner. In a semantic network, information is represented as nodes connected by a set of marked directed lines that represent relationships between nodes (Kang et al., 2017). The assumption behind SNA is a specific connection between the words or concepts that frequently co-occur in the text, along with statistical indicators that can measure these connections. As a result, SNA enables the extraction of important ideas by recognizing emergent clusters of concepts rather than evaluating the frequency of solitary words (Kang et al., 2017; Golob et al., 2018). In this way, we can enhance our understanding of the text of social media users’ comments.

Therefore, with the rapid development of social media, SNA has become one of the commonly used case analysis methods of crisis communication in academia (Schultz et al., 2012; Park et al., 2019). In crisis communication, researchers and practitioners use SNA to infer the cluster composition of words. Then, according to their relevance, the terms are classified into different clusters. Finally, we can extensively explore social media users’ attitudes toward those responsible for the crisis and their main concerns (Scheufele, 1999).

For example, Tang et al. (2018) performed an SNA of people’s comments on Twitter during the measles epidemic of 2015. They found news updates, public health, vaccine, and political clusters and identified different adaptive crisis communications in different stages of the crisis based on these frameworks. Similarly, with the help of SNA, Park et al. (2019) identified that the content framework of tourism organizations dynamically changes in different stages of the crisis. Therefore, we also apply the feature of SNA in the exploration of this case.

### Sentiment Analysis

Sentiment analysis is the process of evaluating, processing, inducing, and reasoning about subjective writings that have emotional hue (Zhao et al., 2020). For example, the Internet produces considerable user engaged, valuable information about people, happenings, goods, and so on. These remarks reflect people’s multiple emotional hues and inclinations, such as joy, rage, grief, criticism, admiration, and so on. As a result, potential users may read these subjective remarks to gain a better understanding of public opinion on a specific incident or item (Kim et al., 2021; Park et al., 2021).

As of 2021, there are two kinds of sentiment analysis methods: sentiment analysis based on lexicon and machine learning (Dhaoui et al., 2017). The lexicon-based sentiment analysis primarily creates a set of sentimental lexicons and rules, performs paragraph deconstruction, parsing, calculates sentimental value, and then utilizes the sentimental value as the foundation of the text’s sentimental tendency (Li and Wang, 2021). Most sentiment analyses based on machine learning translate this challenge into a classification working. The target sentiment is divided into two groups for the purpose of judging sentiment polarity: positive and negative. After manually labeling the training text, a supervised classification procedure is carried out (Bijlstra et al., 2010). For example, machine learning based on large-scale corpus is more common.

Compared with lexicon-based sentiment analysis, machine learning pays less attention to whether the text contains specific sentiment words and relies more on what categories the text is labeled in the training set. The result of machine learning depends on the corpus’s size and the accuracy of corpus annotation. In practice, sentiment analysis based on lexicons performs better in short texts, such as Facebook (Ortigosa et al., 2014), Twitter (Saif et al., 2016), and Sina Weibo (Xue et al., 2014). Meanwhile, the long text is better suited to machine learning. Therefore, this study uses sentiment analysis based on the lexicon.

Sentiment analysis has a good application effect in crisis communication, as it directly presents the two sides of social media users’ comments in the form of scores. For example, Bygstad and Presthus (2013) used Facebook to study customer communication cases of two Scandinavian airlines during the volcanic-ash crisis of April 2010. Their research showed that comparative sentiment analysis of two subjects was adequate to observe the differences in crisis communication strategies.

Sentiment analysis can also evaluate the effect of official crisis communication strategies. For example, Mishra and Sharma (2019) investigate the brand crisis experienced by Nestle’s instant noodle brand Maggi, as well as the extent to which a health-related problem might influence customer response on Facebook for a strong and trusted brand.

Sentiment analysis shows a unique understanding of public attitudes toward crisis communication strategies. Based on Twitter, Kaur et al. (2021) explored the impact of crisis communication strategies of Indian leaders in public health events after the outbreak of COVID-19 on the public’s emotions, which can be divided into 10 types, including anger, expectation, disgust, and fear. Similarly, based on Twitter, Tian et al. (2021) divided sentiments into eight categories, including arousal and valence, and examined the crisis of United Airlines’ in 2017. The authors found that social media sentiment analysis can help identify public responses and help enterprises adopt appropriate communication strategies. Therefore, we also applied the feature of Sentiment analysis to explore this case.

### Situational Crisis Communication Theory

Based on the attribution theory, Coombs created SCCT in 2007, and it has emerged as the most widely used and authoritative Crisis Communication and Management Theory. SCCT says that organizations’ crisis communication strategies should be based on the nature of the crisis. SCCT has three main components: organizational crisis, crisis response strategy, and matching crisis situation and response strategy (Coombs, 2007b).

Some studies have classified communication strategies adopted by organizations using crisis response strategies based on SCCT (Park and Park, 2020) into four types: *deny crisis response strategies*, *diminishing crisis response strategies*, *rebuild crisis response strategies*, *bolstering crisis response strategies* (Coombs, 2007b). As a result, the same work is done in this study, SCCT is used to classify NetEase’s crisis communication tactics.

*Deny crisis response strategies* mainly include following: (a) *Attack the accuser*: crisis managers directly attack those who claim that the organization has problems. (b) *Denial*: the crisis manager asserts that there is no crisis. (c) *Scapegoat*: Other people or organizations are really responsible and insist that there is no crisis.

*Diminishing crisis response strategies* have two main options: (d) *Excuse* means to let the public believe that they have no intention to harm. (e) *Justification* provides rationalized explanations for the occurrence of crisis and reduces the perceived degree of crisis.

*Rebuild crisis Response Strategies include following*: (f) *Compensation* means to compensate the victim in the form of material or money. (g) *Apology* means admitting responsibility and making a frank apology to earn the victim’s or the public’s understanding.

*Bolstering crisis response strategies* focus on strengthening their positive image in various ways, including following: (h) *Reminder*: reminding the public of good experiences in the past, or (i) *Ingratiation*: pleasing stakeholders; (j) *Victimage*: conveying the message that they are also victims.

We qualitatively examined the crisis response strategies NetEase employed in social media from June 6, 2020, right after the incident, to June 9, 2020, as shown in Table 1.

## Materials and Methods

**Figure 1:**
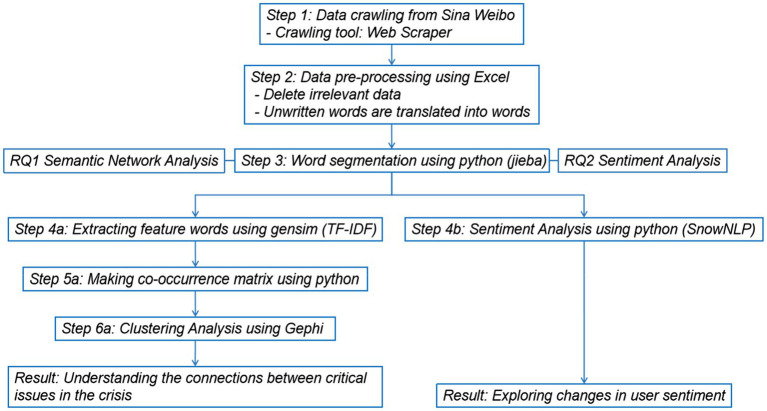
Research method for determining whether NetEase’s crisis communications were successful by combining SNA and sentiment analysis. TF-IDF, term frequency–inverse document frequency; Jieba, a Python package for word segmentation in the Chinese language.

Figure 1 shows the data processing process: how we collected, processed, and analyzed data. We pre-processed the data before answering the RQ: (1) used the Web Scraper to crawl data, (2) used Excel to clean the data, and (3) used Jieba for word segmentation.

SNA was used in RQ1, and the specific process can be divided into following steps: (4a) used Term Frequency–Inverse Document Frequency (TF-IDF) to extract feature words; (5a) obtained a co-occurrence matrix based on feature words; (6a) used Gephi to conduct clustering analysis.

Sentiment lexicon, a type of sentiment analysis, is used in RQ2. Sentiment analysis is divided into machine learning and sentiment lexicon, and the latter is adopted in this study.

### Data Crawling

NetEase issued three apology statements *via* their official Sina Weibo account, eliciting various user responses. To collect these data, we used a web scraper plug-in in Google Chrome, enabling the browser to collect all comments by mimicking human mouse actions. Because the time interval between the three apology announcements was very short and this study only focused on the words under the official account, after data cleaning, we collected 2,066 responses to the first, 912 to the second, and 878 to the third statements (3,856 statements total).

### Data Pre-processing

**Figure 2:**
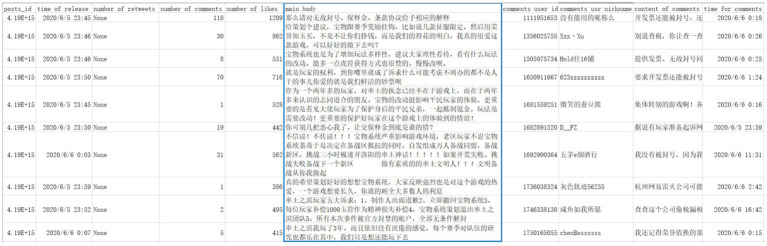
Raw data.

First, we eliminated data not relevant to this study (Che, 2021). As shown in Figure 2, we were only interested in the content of the text (in the blue box), and the rest of the data were deleted, such as posts ID, time of release, etc. Then, using MS Excel, non-essential columns were removed, including timestamps and user nicknames.

Sina Weibo automatically replaces abusive words with emoticons. Hence, we replaced those and user-added emoticons and symbols with emotion-colored textual statements to neutralize the non-textual data as much as possible.

### Word Segmentation

Finally, we used the Jieba python package for word segmentation in Chinese. Jieba supports three modes of segmentation:

*Precise mode* cuts sentence precisely into slices suitable for text analysis.

*Omnibus mode* allows for the faster scanning of words in a sentence.

*Search engine mode* splits longer sentences into smaller ones to improve the recall rate for search engine segmentation.

After several comparisons, the precise mode was selected for this study because the *omnibus mode* does not resolve ambiguity and the *search engine mode* is more suitable for search engine analysis. In contrast, the *precise mode* is the most suitable for text analysis.

### Data Analysis (RQ1 & RQ2)

This study combines SNA and sentiment analysis to measure whether the enterprise’s crisis communication strategy has the expected impact on users’ attitudes. First, SNA was used to conduct a content analysis on comments; then, sentiment analysis was used to calculate the emotional polarity of the comments. For example, suppose the proportion of negative emotions in the comments was much higher than positive ones. In this case, the high-frequency words and cluster analyses generated by SNA would not be positive.

#### SNA

##### Term Frequency-Inverse Document Frequency

We generated a “one-dimensional co-occurrence matrix” using the 50 most important keywords and estimated modularity to gauge the quality of the communities by calculating the term frequency-inverse document frequency (TF-IDF). The TF-IDF method is a statistical analytic method for determining the relative relevance of keywords in document sets or corpora. For example, if a word is somewhat uncommon yet appears frequently in a certain article, it will most likely represent the qualities of the piece.

We generated three datasets of comments (one for each apology statement) and used TF-IDF to extract the most relevant terms from each set. The relevance of a term was related to how many times it appeared in a comment and inversely proportional to how many times it appeared in a corpus. As a result, this computation approach effectively eliminated the effects of common terms on keywords while also improving the association between keywords and articles (Che et al., 2021).

##### Clustering Analysis Using Gephi Through Community Detection Algorithm

We constructed an undirected graph to study the high TF-IDF words in gamer comments. The nodes in the graphs represent common TF-IDF keywords, and the edges indicate the co-occurrence relationship between two nodes. Thus, the nodes’ size reflects the TF-IDF size, and the thickness of the edges reflects the frequency with which the two nodes appear together.

Community detection, a popular method in social network analysis, was utilized to further display nodes and edges. The Louvain algorithm is a modular community finding algorithm. Technique is effective in detecting hierarchical community structures. The detection of communities is accomplished by optimizing the modularity of the overall community network. The degree of tightness inside a community is described by modularity. Because the high degree of modularization reflects the excellent quality division, this study repeated the modular calculation many times to achieve the maximum results. The following is the calculation formula for modularization:


$$\overset{K}{\underset{k=1}{\sum }}\left(e\left(C_{c}^{k},S\right)-a{\left(C_{c}^{k},S\right)}^{2}\right)$$

where $e\left(C_{c}^{k},S\right)$ is the proportion of two vertices of the community edge in community $C_{c}^{k}$ . The term after the formula indicates the proportion of at least one vertex of the edge in community $C_{c}^{k}$ . In a highly modularized network, the connections between nodes are dense, and the connections between nodes in different modules are sparse. A modularity score close to zero indicates that the fraction of edges among communities is no better than random and that close to one indicates that the network community structure is as strong as it can be (Chen et al., 2014).

#### Sentiment Analysis

The emotional tendency can be regarded as a tendency of inner evaluation and liking of the subject to the subjective existence of an object. Two aspects measure it: the direction of emotional inclination and the degree of an emotional preference. As of 2021, sentiment analysis approaches are classified into two types: those based on sentiment lexicons and on machine learning, such as machine learning based on large-scale corpora. The former requires a well-annotated emotional vocabulary, whereas the latter requires a large number of manually annotated corpora as a training set to achieve sentiment classification by extracting text features and building classifiers.

SnowNLP is a popular Python package in Chinese sentiment lexicon analysis in recent 2 years (Huang et al., 2021). Relevant studies have proven that SnowNLP is highly feasible and accurate (Chang et al., 2018; Tseng et al., 2018). It calculates a score per sentence and returns a value between zero and one, and it is used for sentiment analysis to determine the emotional polarity of comments. A score closer to one indicates a positive emotion, whereas a score closer to zero indicates a more negative emotion, and middling scores indicate neutral emotions. Sentiment analysis was used in this study to measure the impact of enterprises’ crisis communication strategies on users.

## Results

### Term Frequency-Inverse Document Frequency

**Figure 3:**
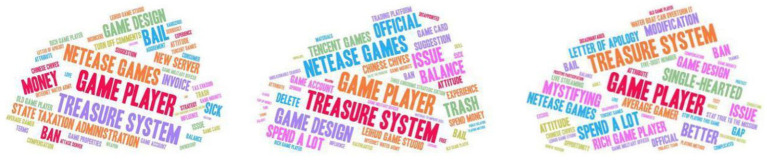
Term frequency–inverse document frequency word clouds of responses to the apology announcements.

In this study, we used the Gensim library in Python to calculate the TF-IDF. Figure 3 shows the top 50 words in word cloud form. Table 2 shows the top 10 words.

**Table 2:** Term frequency–inverse document frequency scores of responses to the apology announcements.

First apology		Second apology		Third apology	
Keyword	Value	Keyword	Value	Keyword	Value
Game player	660	Game player	141	Game player	261
Treasure system	520	Treasure system	136	Treasure system	227
NetEase games	413	NetEase Games	108	spend a lot	125
bail	368	game design	92	better	122
money	333	issue	80	single-hearted	116
game design	318	official	79	mystifying	113
ban	286	trash	72	issue	107
sick	219	balance	65	NetEase Games	105
sta	208	spend a lot	57	money-rich	94
invoice	195	Tencent Games	57	ban	94

sta stands for state taxation administration.

We can see from Figure 3 and Table 2 that Game player and Treasure system are consistently the most critical core words in the three reviews. Secondly, each comment data set is represented by specific terms, such as bail, money, and sta in the first data set; Issue, official, and trash in the second dataset; Better, single-hearted, and mystifying in the third dataset.

### Clustering Analysis Using Gephi Through Community Detection Algorithm

In this study, GePhi0.9.2 was used to retrieve word clusters through a community detection algorithm and visualization technique. The layout algorithm selected was “Fruchterman Reingold,” for which the sizes of nodes and labels are determined according to their degree of centrality. The larger the degree of centrality, the larger the nodes and labels. In addition, the co-occurrence frequency determines the connection thickness between nodes. This study used the modularity function of Gephi, which has a built-in Louvain algorithm. Finally, nodes of the same color represent the same community.

Table 3 shows the percentages of each cluster for the three communications. Finally, we manually assigned the specific cluster description based on the text, as shown in Table 4.

**Table 3:** Percentages of each community according to the three communications.

Network	Edges	Clusters	1st cluster	2nd cluster	3rd cluster
1	594	3	60%	28%	12%
2	328	3	38%	34%	28%
3	420	3	54%	40%	6%

**Table 4:** Cluster descriptions.

**1st apology**	**Cluster description**
Cluster 1	Players are unhappy with the way NetEase is handling the issue
Cluster 2	Treasure system breaks the balance of the game
Cluster 3	Account suspension is illegal
**2nd apology**	**Cluster description**
Cluster 1	Players are unhappy with the way NetEase is handling the issue
Cluster 2	Treasure system breaks the balance of the game
Cluster 3	Players demanded the Treasure system be removed
**3rd apology**	**Cluster description**
Cluster 1	Players are unhappy with the way NetEase is handling the issue
Cluster 2	Treasure system breaks the balance of the game
Cluster 3	Players see this as an opportunity for NetEase

#### First Apology Statement

**Figure 4:**
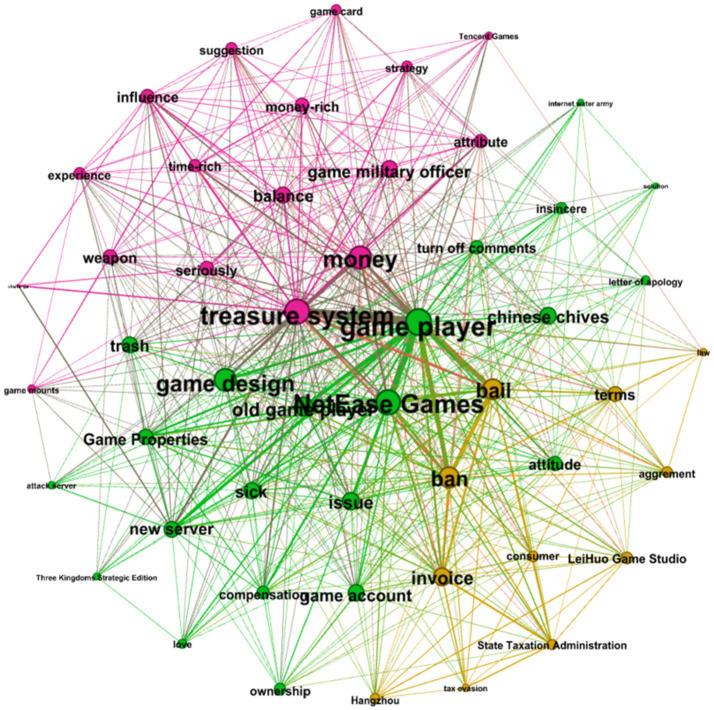
Semantic network of user reviews after the first apology statement by NetEase.

As the cluster in Figure 4 shows, the green network accounted for 60% of the total comments, and it describes players’ dissatisfaction with punishments for game users and requirement for bail money to unlock accounts. Players further expressed their protest plans through various actions. “Sick,” “trash,” and “attitude” refer to the players’ attitude toward IC. “New servers” and “attack servers” refer to groups of players threatening or planning to attack the game’s servers, and “closed comments” refer to IC removing negative user comments.

The purple network (28%) describes how players felt about the “Treasure system,” with the keywords “game cards” and “balance” referring to the game’s new version disrupting the game’s content and balance, respectively.

The brown network (12%) refers to players who considered IC’s actions to have seriously violated the law. For example, players asked the State Taxation Administration to investigate IC because IC refused to issue receipts for the bail that IC asked players to pay.

#### Second Apology Statement

**Figure 5:**
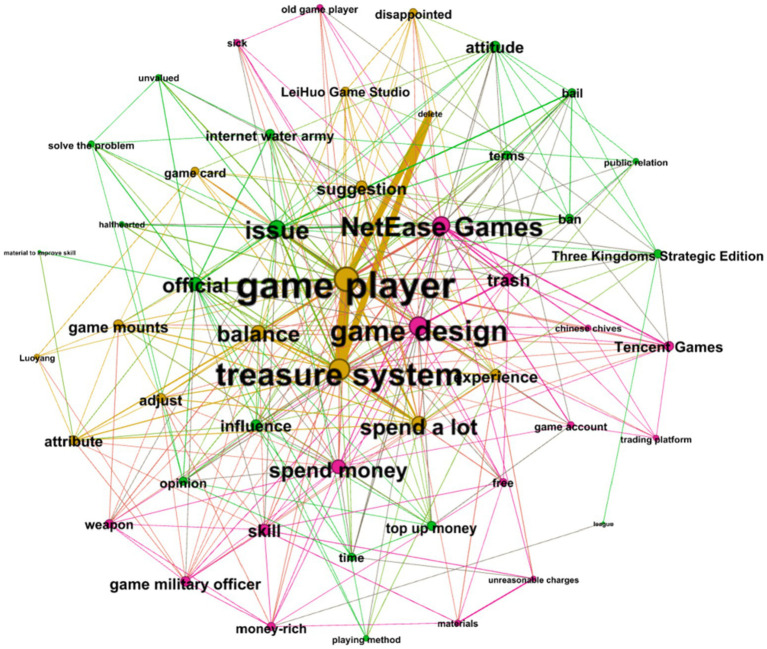
Semantic network of user reviews after the second apology statement by NetEase.

In the cluster shown in Figure 5, the brown network accounted for 28% of comments. Although not the most crucial cluster, this cluster has the most central node. From “Game player,” “Treasure system,” and “delete,” we can see that the Game players asked IC to remove the Treasure system.

The green network accounted for 38% of comments, showing players’ dissatisfaction with IC’s handling, including words like “handle,” “attitude,” “fine,” “half-hearted,” and “protest.” The purple network (34%) describes players who feel the Treasure system breaks the game’s balance and are dissatisfied with the game’s design and designers.

#### Third Apology Statement

**Figure 6:**
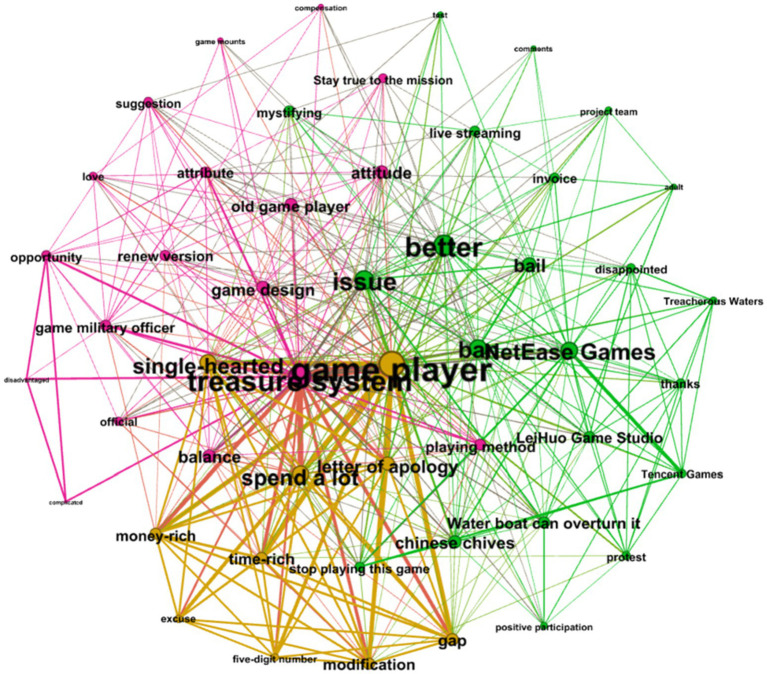
Semantic network of user reviews after the third apology statement by NetEase.

As Figure 6 shows, the brown networks account for 40% of the comments. Players say the Treasure system breaks the game’s balance, widens the gap between money-rich and time-rich players, and makes it harder for ordinary players to enjoy the game.

The green network (54%) showed that Game players still expressed dissatisfaction with how the IC handled the problem. “Water can overturn a boat” is a Chinese saying that suggested IC to re-examine its relationship with its users.

In the purple network (6%), we found users advising IC to use this opportunity to improve its products, services, and relationships with customers.

### Sentiment Dictionary Analysis

We further trained the data on the Jieba dictionary, manually adding 250 positive and 250 negative texts.

#### Overall Sentiment Proportion and Change Trend

**Figure 7:**
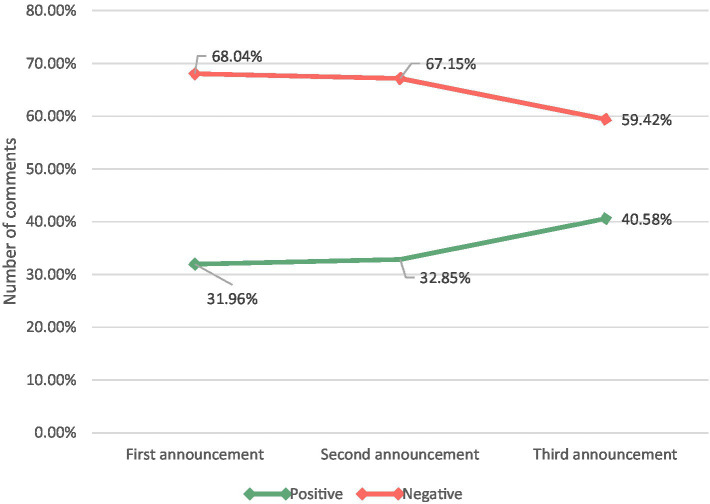
Overall emotional percentage change graph.

As shown in Figure 7 and Table 5, the proportions of positive emotions corresponding to the first, second, and third apologies was 31.96, 32.85, and 40.58%, respectively. Conversely, the proportions of negative emotions to the first, second, and third apology statements were 68.04, 67.15, and 59.42%, respectively. Thus, overall, the proportion of positive comments gradually increased, and there was almost no change between the second and the first; the third apology showed an apparent upward trend.

**Table 5:** Ratio of positive and negative emotions in three comments.

Score	1st time		2nd time		3rd time	
	Volume	Ratio	Volume	Ratio	Volume	Ratio
0 ≤ score ≤ 0.5 (negative)	1,406	68.04%	612	67.15%	522	59.42%
0.5 < score ≤ 1.0 (positive)	660	31.96%	300	32.85%	356	40.58%

The second official announcement did not work well as it did not address the players’ demands, whereas the third official announcement addressed some problems.

#### Sentiment Proportion and Change Trend in the Unit of Score Interval

This study divided the range (0–1) into five smaller intervals to observe the changes in sentiment values at smaller gaps.

As shown in Table 6, the emotional scores of the first apology were 46.61, 13.89, 14.67, 08.52, and 16.31%, respectively. The emotional scores of the second were 42.76, 16.24, 14.58, 11.73, and 14.70%, respectively. The emotional scores for the third were 35.99, 16.97, 13.90, 14.12, and 19.02%, respectively.

**Table 6:** Segmentation of the emotional proportion of three comments.

Score	1st apology		2nd apology		3rd apology	
	Volume	Ratio	Volume	Ratio	Volume	Ratio
0.0 ≤ score ≤ 0.2	963	46.61%	390	42.76%	316	35.99%
0.2 < score ≤ 0.4	287	13.89%	148	16.24%	149	16.97%
0.4 < score ≤ 0.6	303	14.67%	133	14.58%	122	13.90%
0.6 < score ≤ 0.8	176	08.52%	107	11.73%	124	14.12%
0.8 < score ≤ 1.0	337	16.31%	134	14.70%	167	19.02%

As shown in Figure 7, the first two statements of NetEase were dominated by words that reflected negative community responses. The negative emotions accounted for a high proportion of sentiment, for which explanations and excuses were near 50%. However, the positive sentiment significantly increased after the avoidance of explanatory words in the third statement.

## Discussion

### RQ1: What Were the Reactions of Users After NetEase Issued Three Statements?

To have a more intuitive understanding of the changes in the content discussed by users, we made the topic change diagram.

**Figure 8:**
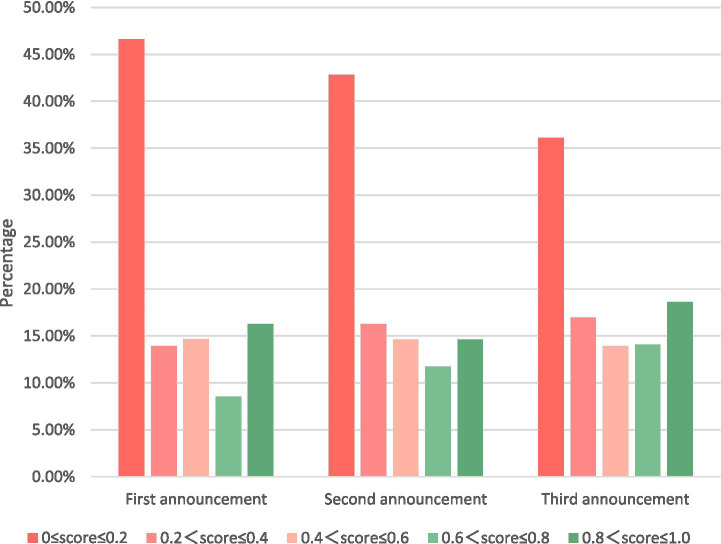
Sentiment changes.

As shown in Figure 8, we found that the clusters (represented by the green line) spanned three review datasets, implying that IC’s three-time strategy (*excuse*, *justification*, *and apology & compensation*) did not resolve players’ persistent dissatisfaction with the way IC handled the incident.

The purple line showing an upward trend demonstrates that gamers were paying more attention to game content. This meant that IC’s third strategy (*apology & compensation*) may gradually directed the player’s attention to the game itself. No matter how much IC tried to explain the purpose of the Treasure system, the Treasure system was still perceived as unbalanced.

The legal action clusters (represented by the red line) appeared only in the first dataset. This indicates that the *diminishing (justification) communication strategy* adopted by IC in the second apology successfully reduced or diverted the attention of Game players from creating a legal issue.

The clusters represented by the blue line appeared only in the third dataset. This reveals that the *rebuild (apology & compensation) crisis communication strategy* adopted by IC in the third apology positively impacted users’ attitudes. IC succeeded in making the Game players hope for the future of IC after the third apology.

### RQ2: How Did Users’ Feelings Change After NetEase Released Each Statement?

**Figure 9:**
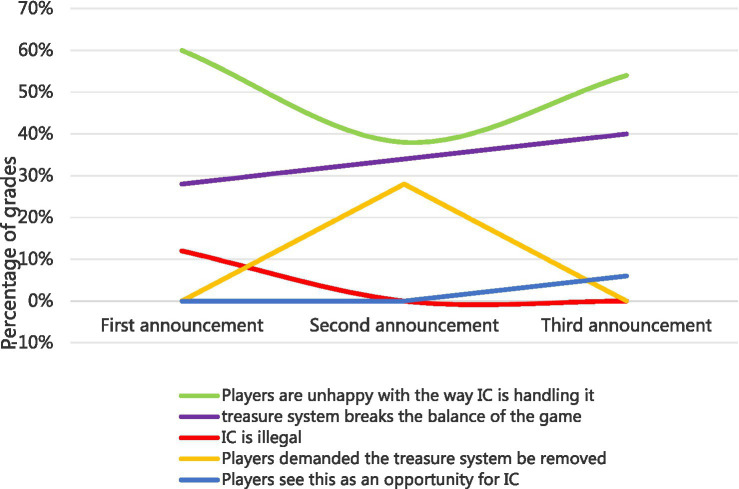
Clusters changing over time.

As shown in Figure 7, the overall negative emotions (0 ≤ score ≤ 0.5) in the first two datasets barely changed (68.04% ≈ 67.15%), which may indicate that the *diminishing (justification) crisis communication strategies* adopted by IC in the second announcement was ineffective. However, Figure 9 shows that extreme negative sentiment (0 ≤ score ≤ 0.2) decreased slightly (46.62% → 42.82%), whereas milder negative sentiment (0.2 < score ≤ 0.4) increased slightly (13.92% → 16.27%). This result shows that although the *Diminishing (justification) communication strategy* adopted by IC for the second announcement was not effective, it still alleviated the more extreme expressions and appeals of Game players.

After IC proposed specific compensation measures in the third apology, the overall positive sentiment (0.5 < score ≤ 1.0) increased significantly (32.85% → 40.58%), indicating that IC’s communication strategy in the third apology (*Rebuild (apology & compensation) crisis communication strategy*) might have a positive impact on player attitudes.

Overall, the *diminishing crisis communication strategies (excuse and justification)* did not change players’ negative attitudes, so they were not successful. But it was not a failure because it successfully defused the player’s legal complaint and focused the player’s attention on the game itself. Finally, the *rebuild (Apology & Compensation)* strategy was the most successful strategy because it significantly increased the percentage of positive emotions and regenerated expectations for IC.

### Notable Findings

For the first time, the litigation crisis was identified within gamer communications with respect to Chinese gaming companies.

“请你对我们所有违法行为做出解释，包括所谓的保释金.”

Translation: “Please account for all our violations, including the so-called bail money.”

Nevertheless, this does not indicate an increase in overall legal awareness among the larger Chinese population. It may only reflect greater legal awareness among Chinese online gamers. For example, Ma et al. found that Chinese people’s legal awareness of food safety was weak (Ma et al., 2020). Perhaps Chinese people have different legal awareness in various fields; this requires further research and observation.

In all three comments, “attitude” was at the forefront of the TF-IDF high-frequency word sets. By returning to the original text, we found that Game players believed that NetEase had a poor attitude toward their crisis communication process and that users were not treated well, as shown below:

“就这?这就是你们的态度?就拖呗，避重就轻呗.”

Translation: “Is this it? Is that your attitude? Just procrastinate, avoid the most important things.”

Game players desired “an equal relationship between game companies and their players.” However, NetEase failed to balance the relationship between the enterprise and its users, resulting in the phrase “Tencent Games” to appear in numerous comments. Tencent Games is NetEase’s biggest competitor in the Chinese market. Bolton et al. demonstrated that consumers would compare their earnings with corporate profits in a market environment and oppose unfair systems. If the pricing looks greedy and results in higher profits for the company than in consumer satisfaction, they may choose to avoid the product and instead buy products from competitors (Bolton et al., 2003).

## Conclusion

This research considers the crisis events of NetEase Games’ game “Immortal Conquest” in the Chinese market in 2020 as the research object. Based on the SCCT, we used SNA and sentiment analysis to understand user feedback content and discussed how the corporate crisis communication strategy affects users’ attitudes.

First, our results demonstrate that the *diminishing crisis communication (excuse and justification)* strategies did not change players’ negative attitudes. Still, it was not a failure because it successfully defused the player’s legal complaint and focused the player’s attention on the game itself.

Second, the *rebuild (apology & compensation) strategy* was effective because it significantly increased the percentage of positive emotions and regenerated expectations for the game.

Third, the litigation crisis was identified within gamer communications with respect to Chinese gaming companies for the first time. Nevertheless, this does not indicate an increase in overall legal awareness among the larger Chinese population. It may only reflect greater legal awareness among Chinese online gamers. This requires further research and observation.

Fourth, gamers emphasize that they and enterprises should be equal when communicating, because we found that NetEase failed to balance the relationship between the enterprise and its users, and Game players believed that NetEase had a poor attitude toward their crisis communication process and that users were not treated well.

Fifth, as the data indicated in-game paid items should be reasonably priced. Otherwise, they will drive users to competitors.

Finally, this study suggests that after the outbreak of a crisis, the online game enterprises in crisis communication can use SNA first to understand the needs of users and the connection between critical issues in the crisis. Secondly, sentiment analysis is used to assess the effectiveness of the communication strategy, according to the validity used to adjust their communication strategy, finally used to achieve the goal of appeasing the crisis.

## Limitations and Future Research

First, given that users’ emotions and demands can vary depending on age or gender, it may be meaningful to consider the age and gender of users when evaluating the crisis communication process.

Second, future studies need to evaluate the process of crisis communication in other countries and cultures using sentiment analysis and SNA.

Third, we replaced visual emoticons with words. While we minimized the impact of substitution, the effect of this behavior on the text is unmeasured. Therefore, we should strengthen the processing of visual emoticons in the future word processing of Sina Weibo (Sion, 2019).

Fourth, the relationship between social media content (Taylor et al., 2020), users’ emotions and demands, and mobile game addiction in terms of semantic network and sentiment analysis has not been covered (Mircică, 2020; Pop et al., 2021).

Finally, the relationship between social media crisis communications and visual content-sharing applications in terms of semantic network and sentiment analysis has not been covered (Bratu, 2019; Adams et al., 2020).

## Data Availability Statement

The original contributions presented in the study are included in the article/supplementary material, and further inquiries can be directed to the corresponding author.

## Author Contributions

SC: conceptualization, research design, implementation, writing, and editing. DN: conceptualization, writing, and editing. PK and SZ: writing and editing. JK: conceptualization, research design, writing, and editing. All authors contributed to the article and approved the submitted version.

## Funding

This work was supported by a National Research Foundation of Korea (NRF) grant funded by the Korean government (NRF-2020R1A2C1014957).

## Conflict of Interest

The authors declare that the research was conducted in the absence of any commercial or financial relationships that could be construed as a potential conflict of interest.

## Publisher’s Note

All claims expressed in this article are solely those of the authors and do not necessarily represent those of their affiliated organizations, or those of the publisher, the editors and the reviewers. Any product that may be evaluated in this article, or claim that may be made by its manufacturer, is not guaranteed or endorsed by the publisher.
